# Supplementary material for: Cunninghamella arunalokei a New Species of Cunninghamella from India Causing Disease in an Immunocompetent Individual
Source: J Fungi (Basel). 2021 Aug 19;7(8):670. doi: 10.3390/jof7080670 (PMC8401845; doi:10.3390/jof7080670)
Supplement: Supplementary file 1 [file jof-07-00670-s001.zip › jof-1290227-supplementary.pdf]

Journal of *Fungi*

MDPI

Supplementary Material

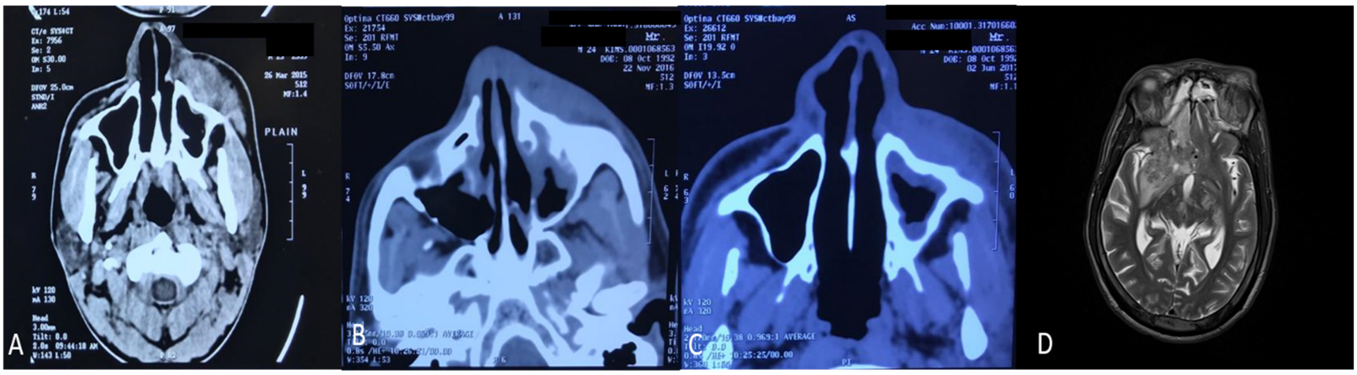

Figure S1. Radiological progression of the lesion over the five years (**A**) lesion in the skin and subcutaneous tissue of left side of the face (2015) (**B, C**) progression with involvement of the left maxillary sinus mucosa thickening (2016 & 2017) & (**D**) Intracranial extension in 2021.

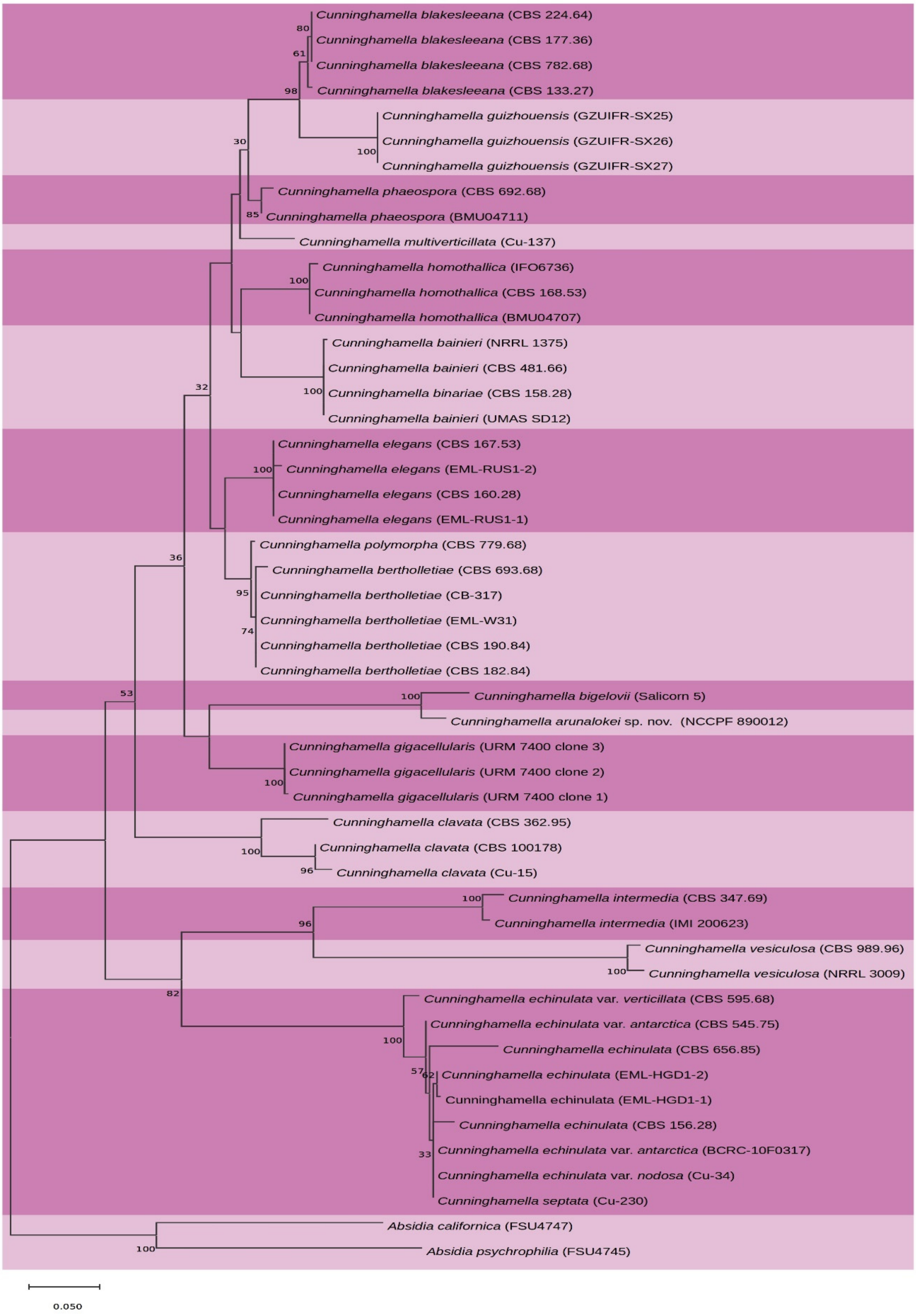

Figure S2: Molecular phylogenetic analysis of *Cunninghamella* species using internal transcribed spacer region The evolutionary history was inferred by using the Maximum Likelihood method based on the Kimura 2-parameter model. All positions containing gaps and missing data were eliminated.

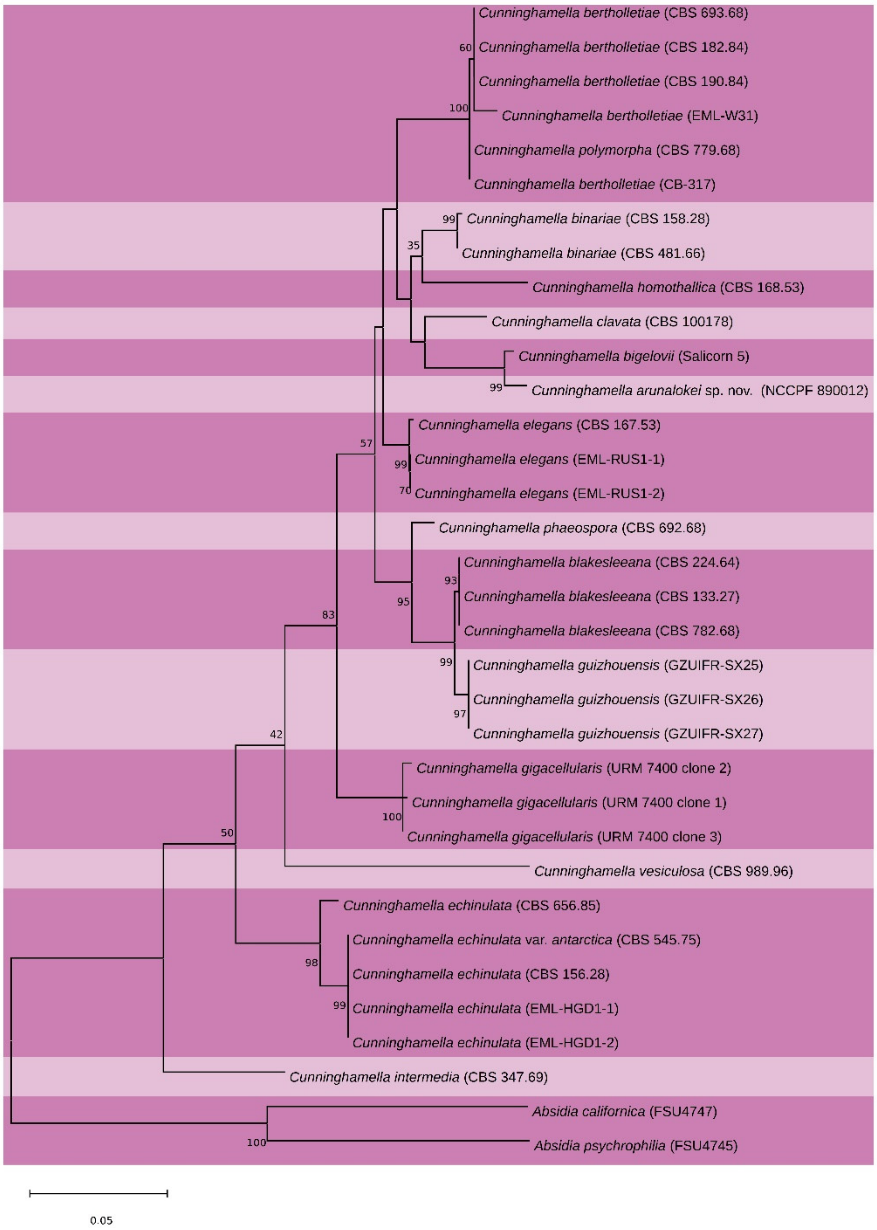

Figure S3: Molecular phylogenetic analysis of *Cunninghamella* species using 28S large ribosomal subunit region. The evolutionary history was inferred by using the Maximum Likelihood method based on the Kimura 2-parameter model. All positions containing gaps and missing data were eliminated.

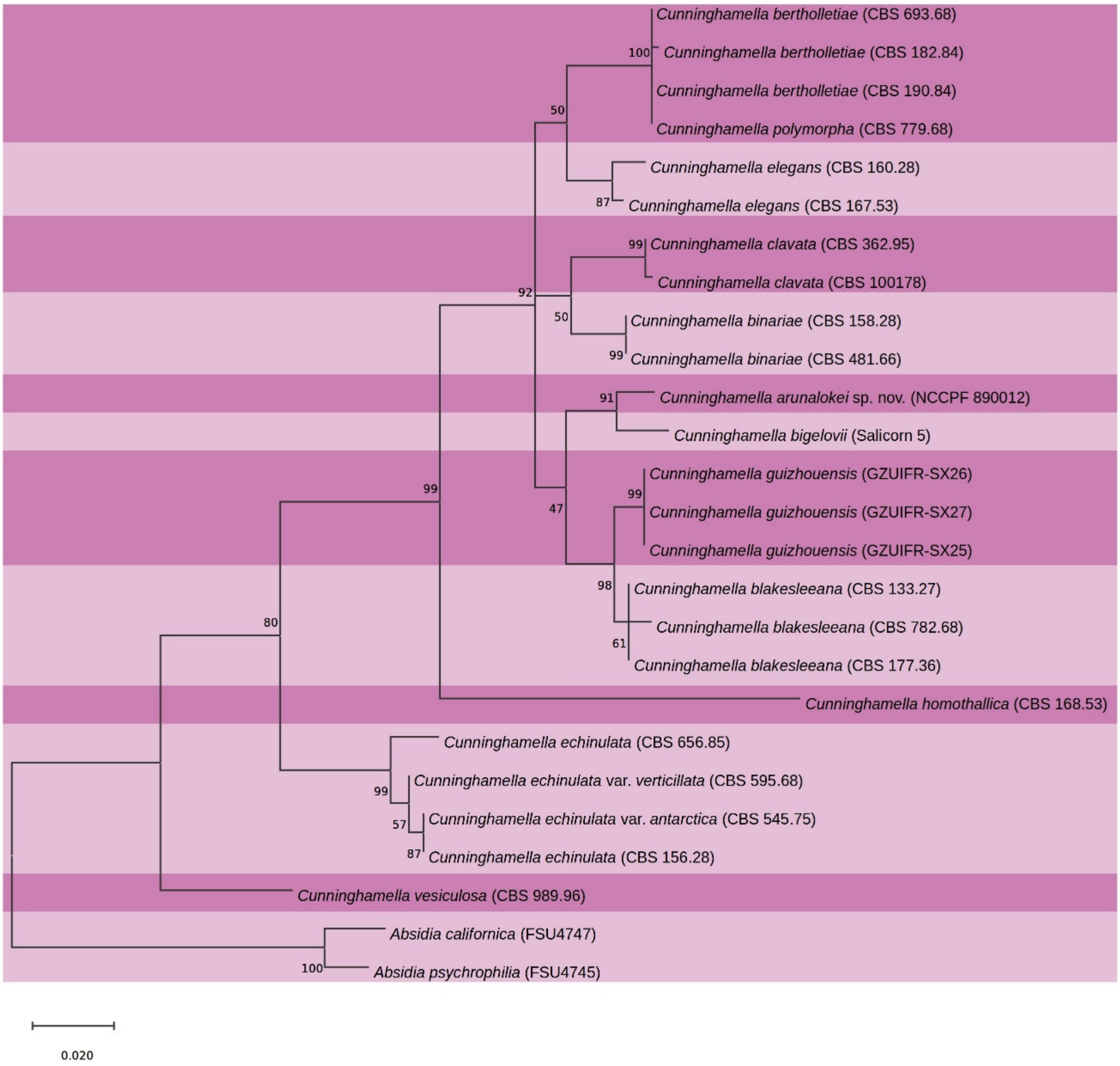

Figure S4. Molecular phylogenetic analysis of *Cunninghamella* species using EF-1 alpha region. The evolutionary history was inferred by using the Maximum Likelihood method based on the Kimura 2-parameter model. All positions containing gaps and missing data were eliminated.

Table S1. Accession numbers of the internal transcribed spacer (ITS) region, large ribosomal subunit (28S) and translation elongation factor-1 alpha (EF-1alpha) of the various *Cunninghamella* species used for phylogenetic analysis in this study.

S. No	Species	Strain ID	ITS	28S	EF-1alpha
1	*C. bainieri*	NRRL 1375	AF254935.1	NA	NA
2	*C. bainieri*	UMAS SD12	KF201293.1	NA	NA
3	*C. bertholletiae*	CB-317	KX638508.1	KX638507.1	NA
4	*C. bertholletiae*	CBS 182.84	JN205877.1	MH873434.1	KJ156481.1
5	*C. bertholletiae*	CBS 190.84	JN205878.1	HM849701.1	KJ156485.1
6	*C. bertholletiae*	CBS 693.68	AF254931.1	MH870924.1	KJ156490.1
7	*C. bertholletiae*	EML-W31	MF806022.1	MF806029.1	NA
8	*C. bigelovii*	Salicorn 5	KJ013403.1	KJ013405.1	KJ395944.1
9	*C. echinulata*	EML-HGD1-2	MF806024.1	MF806026.1	NA
10	*C. echinulata* var. *antarctica*	CBS 545.75	AF254938.1	JN206597.1	KJ156492.1
11	*C. elegans*	EML-RUS1-2	MF806021.1	MF806028.1	NA
12	*A. psychrophilia*	FSU4745	AY944874.1	EU736306.1	EU736252.1
13	*A. californica*	FSU4747	AY944872.1	EU736300.1	EU736246.1
14	*C. arunalokei*	IL3459	MN431159.1	MN431158.1	This study
15	*C. binariae*	CBS 158.28	JN205888.1	JN206602.1	KJ156486.1
16	*C. blakesleeana*	CBS 133.27	AF254932.1	NG_063968.1	KJ156479.1
17	*C. blakesleeana*	CBS 224.64	JN205870.1	MH870055.1	NA
18	*C. blakesleeana*	CBS 177.36	JN205868.1	NA	KJ156483.1
19	*C. blakesleeana*	CBS 782.68	JN205869.1	JN206601.1	KJ156478.1
20	*C. clavata*	CBS 100178	JN205890.1	HM849696.1	KJ156477.1
21	*C. clavata*	CBS 362.95	JN205891.1	NA	KJ156473.1
22	*C. clavata*	Cu-15	AF254942.1	NA	NA
23	*C. echinulata*	CBS 156.28	JN942997.1	JN939199.1	KJ156500.1
24	*C. echinulata*	CBS 656.85	JN942996.1	JN939200.1	KJ156491.1
25	*C. echinulata*	EML-HGD1-1	MF806020.1	MF806025.1	NA
26	*C. echinulata* var. *antarctica*	BCRC-10F0317	JQ678764.1	NA	NA
27	*C. echinulata* var. *nodosa*	Cu-34	AF346407.1	NA	NA
28	*C. echinulata* var. *verticillata*	CBS 595.68	AF254937.1	NA	KJ156475.1
29	*C. elegans*	CBS 167.53	JN205882.1	HM849700.1	KJ156494.1
30	*C. elegans*	CBS 481.66	JN205889.1	JN206603.1	KJ156495.1
31	*C. elegans*	EML-RUS1-1	MF806023.1	MF806027.1	NA
32	*C. elegans*	CBS 160.28	AF254928.1	NA	KJ156470.1
33	*C. gigacellularis*	URM 7400 clone 1	KX238886.1	KX238889.1	NA
34	*C. gigacellularis*	URM 7400 clone 2	KX238887.1	KX238890.1	NA
35	*C. gigacellularis*	URM 7400 clone 3	KX238888.1	KX238891.1	NA
36	*C. guizhouensis*	GZUIFR-SX25	MN908596.1	MN908599.1	MN912633.1
37	*C. guizhouensis*	GZUIFR-SX26	MN908597.1	MN908600.1	MN912634.1
38	*C. guizhouensis*	GZUIFR-SX27	MN908598.1	MN908601.1	MN912635.1
39	*C. homothallica*	CBS 168.53	JN205863.1	JN206605.1	KJ156498.1
40	*C. homothallica*	BMU04707	MN217567.1	NA	NA
41	*C. homothallica*	IFO6736	AF254941.1	NA	NA
42	*C. intermedia*	CBS 347.69	AF254940.1	JN206606.1	NA
43	*C. intermedia*	IMI 200623	AF254939.1	NA	NA
44	*C. multiverticillata*	Cu-137	AF254933.1	NA	NA
45	*C. phaeospora*	BMU04711	MG583991.1	NA	NA
46	*C. phaeospora*	CBS 692.68	AF254934.1	HM849697.1	NA

47	*C. polymorpha*	CBS 779.68	JN205874.1	JN206599.1	KJ156471.1
48	*C. septata*	Cu-230	AF346408.1	NA	NA
49	*C. vesiculosa*	CBS 989.96	JN205897.1	HM849693.1	KJ156474.1
50	*C. vesiculosa*	NRRL 3009	AF254943.1	NA	NA

Table S2. Details of various Mucorales genomes used for whole-genome phylogeny in this study.

Name	Strain	NCBI Genome Accession	SRA ID	SRX ID
A. glauca	CBS 101.48 substr. RVII-324 met-	GCA_900079185.1	-	-
A. repens	NRRL 1336	GCA_002105175.1	-	-
A. elegans	B7760	GCA_000696995.1	-	-
A. ossiformis	NRRL A-21654	GCA_014839865.1	-	-
A. trapeziformis	B9324	GCA_000696975.1	-	-
A. variabilis	NCCPF 102052	GCA_002749535.1	-	-
C. cucurbitarum	KUS-F28377	GCA_001683725.1	-	-
C. recurvatus	B5483	GCA_000697235.1	-	-
C. antarctica	NRRL 6534	-	SRR7686432	SRX4546928
C. arunalokei sp. nov.	IL3459	This study	This study	This study
C. bertholletiae	175	GCA_000697215.1	-	-
C. bertholletiae	B7461	GCA_000697315.1	-	-
C. bertholletiae	NRRL 1380	-	SRR9718426	SRX6476271
C. bertholletiae	NRRL 1377	-	SRR9719771	SRX6477469
C. echinulata	RSA 2017+	-	SRR10125925	SRX6854350
C. echinulata	RSA 2018-	-	SRR10125928	SRX6854353
C. echinulata	NRRL 1385	-	SRR9719880	SRX6477578
C. echinulata	NRRL 1386	-	SRR9720690	SRX6478239
C. echinulata	NRRL 1387	-	SRR9721137	SRX6478686
C. elegans	NRRL 1392	-	SRR9719892	SRX6477590
C. elegans	NRRL 1388	-	SRR9719893	SRX6477591
C. elegans	NRRL 1390	-	SRR9719896	SRX6477594
C. elegans	NRRL 1391	-	SRR9719906	SRX6477604
C. elegans	NRRL 1393	-	SRR9719909	SRX6477607
C. polymorpha	NRRL 6428	-	SRR12691406	SRX9171364
Gigaspora rosea	DAOM 194757	GCA_003550325.1	-	-
Glomus cerebriforme	DAOM 227022	GCA_003550305.1	-	-
Gongronella sp.	w5	GCA_001650995.1	-	-
Hesseltinella vesiculosa	NRRL 3301	GCA_002104935.1	-	-
Lichtheimia corymbifera	FSU 9682	GCA_000723665.1	-	-
Lichtheimia ramosa	KPH11	GCA_008728235.1	-	-
Linnemannia elongata	AG-77	GCA_001651415.1	-	-
Mortierella alpina	ATCC 32222	GCA_000240685.2	-	-
Mortierella hygrophila	NRRL 2591	GCA_015680625.1	-	-
Mucor ambiguus	NBRC 6742	GCA_000950595.1	-	-
Mucor circinelloides	JCM 22480	GCA_001599575.1	-	-
Mucor irregularis	B50	GCA_000587855.1	-	-
Mucor lusitanicus	MU402	GCA_010203745.1	-	-
Mucor racemosus	B9645	GCA_000697255.1	-	-
Mucor velutinosus	B5328	GCA_000696895.1	-	-

Phycomyces blakesleeanus	NRRL 1555(-)	GCA_001638985.2	-	-
Podila verticillata	NRRL 6337	GCA_000739165.1	-	-
Rhizomucor miehei	CAU432	GCA_000611695.1	-	-
Rhizomucor pusillus	-	GCA_900175165.2	-	-
Rhizophagus irregularis	DAOM 197198	GCA_000439145.3	-	-
Rhizopus azygosporus	CBS 357.93	GCA_003325435.1	-	-
Rhizopus delemar	RA 99-880	GCA_000149305.1	-	-
Rhizopus microsporus	ATCC 52813	GCA_002708625.1	-	-
Rhizopus oryzae	99-892	GCA_000697725.1	-	-
Rhizopus stolonifer	B9770	GCA_000697035.1	-	-
Saksenaea oblongispora	B3353	GCA_000697495.1	-	-
Saksenaea vasiformis	B4078	GCA_000697055.1	-	-
Syncephalastrum contaminatum	UoMD18-1	GCA_013461545.1	-	-
Syncephalastrum monosporum	B8922	GCA_000697355.1	-	-
Syncephalastrum racemosum	NRRL 2496	GCA_002105135.1	-	-
Thermomucor indicae-seudaticae	HACC 243	GCA_000787465.1	-	-
